# A Novel QTL for Powdery Mildew Resistance in Nordic Spring Barley (*Hordeum vulgare* L. ssp. *vulgare*) Revealed by Genome-Wide Association Study

**DOI:** 10.3389/fpls.2017.01954

**Published:** 2017-11-14

**Authors:** Therése Bengtsson, Inger Åhman, Outi Manninen, Lars Reitan, Therese Christerson, Jens Due Jensen, Lene Krusell, Ahmed Jahoor, Jihad Orabi

**Affiliations:** ^1^Department of Plant Breeding, Swedish University of Agricultural Sciences, Uppsala, Sweden; ^2^Boreal Plant Breeding Ltd., Jokioinen, Finland; ^3^Graminor AS, Ridabu, Norway; ^4^Lantmännen Lantbruk, Malmö, Sweden; ^5^Nordic Seed A/S, Galten, Denmark; ^6^Sejet Plant Breeding, Horsens, Denmark

**Keywords:** *Blumeria graminis* f. sp. *hordei*, *mlo*, GWAS, *Hordeum vulgare* L., linkage disequilibrium, plant breeding, resistance

## Abstract

The powdery mildew fungus, *Blumeria graminis* f. sp. *hordei* is a worldwide threat to barley (*Hordeum vulgare* L. ssp. *vulgare*) production. One way to control the disease is by the development and deployment of resistant cultivars. A genome-wide association study was performed in a Nordic spring barley panel consisting of 169 genotypes, to identify marker-trait associations significant for powdery mildew. Powdery mildew was scored during three years (2012–2014) in four different locations within the Nordic region. There were strong correlations between data from all locations and years. In total four QTLs were identified, one located on chromosome 4H in the same region as the previously identified *mlo* locus and three on chromosome 6H. Out of these three QTLs identified on chromosome 6H, two are in the same region as previously reported QTLs for powdery mildew resistance, whereas one QTL appears to be novel. The top NCBI BLASTn hit of the SNP markers within the novel QTL predicted the responsible gene to be the 26S proteasome regulatory subunit, RPN1, which is required for innate immunity and powdery mildew-induced cell death in *Arabidopsis*. The results from this study have revealed SNP marker candidates that can be exploited for use in marker-assisted selection and stacking of genes for powdery mildew resistance in barley.

## Introduction

Powdery mildew is one of the major diseases of barley (*Hordeum vulgare* L.) caused by the powdery mildew fungus, *Blumeria graminis* f. sp. *hordei*, an obligate biotrophic pathogen (Glawe, 2008). Control of powdery mildew may be achieved by fungicide applications, by deployment of resistant cultivars or a combination of the two. The use of host plant resistance is an environmentally sound alternative; however introduction of single resistance genes often leads to the so called ‘boom and bust cycles,’ where cultivars with a single major resistance gene are grown extensively until the pathogen overcomes the resistance. One way to avoid the ‘boom and bust cycles’ is to grow cultivars with broad-spectrum resistance, effective against several or all pathotypes. A successful example of this in barley is the use of the loss-of-function (*mlo)* alleles of the *Mlo* gene on chromosome 4H which has led to nearly complete resistance to powdery mildew, durable for more than 50 years (Jørgensen, 1992; Büschges et al., 1997). Before *mlo* was deployed, barley breeders made efforts to combine major resistance genes to combat the prevailing pathotypes. The locations of several major genes and quantitative trait loci (QTLs) associated with powdery mildew resistance have now been identified in barley (Friedt and Ordon, 2007). For example, major resistance genes have been mapped to chromosome 1H (*Mla, Mlat, Mlk, Mlnn, Mlra, MlGa*, and *Mlp*), 2H (*MlLa*), 4H (*Mlg)*, 5H (*Mlj*), 6H (*Mlh*), and 7H (*mlt* and *Mlf*) (Jensen and Jørgensen, 1975; Doll and Jensen, 1986; Hilbers et al., 1992; Görg et al., 1993; Jørgensen and Wolfe, 1994; Schönfeld et al., 1996; Wei et al., 1999).

To enable the breeding of disease resistant barley material while at the same time maintain and/or improve important agronomical traits such as yield and malting quality, it is important to dissect the genetics behind the resistance traits. One method to localize genes for important agronomical traits is genome-wide association study mapping (GWAS) also known as linkage disequilibrium (LD) mapping (Zhu et al., 2008). GWAS takes advantage of ancestral recombination events to identify significant phenotypic and genotypic associations. One of the advantages of GWAS, compared to traditional linkage mapping of populations from bi-parental crosses, is that alleles present within diverse sets of accessions or existing natural populations can be identified (Zhu et al., 2008). However, to avoid false positives, a limit for the minor allele frequency (MAF) needs to be set, something which restricts the chance to detect rare and potentially unexploited alleles (Tabangin et al., 2009). The power of the GWAS lies in the degree of LD between the marker allele and the functional disease resistance allele. In cultivated barley, LD between markers has been found to be extensive, from 1 cM to beyond 10 cM (Kraakman et al., 2004; Caldwell et al., 2006; Malysheva-Otto et al., 2006; Rostoks et al., 2006; Comadran et al., 2011; Bengtsson et al., 2017). Compared to wild barley (*H. vulgare* ssp. *spontaneum* C. Koch) populations, the LD in elite breeding germplasm extends much further (Caldwell et al., 2006). Another important factor to consider when conducting GWAS analyses is the population structure (genetic relatedness) within the set of accessions, since it can inflate the number of spurious marker-trait associations identified (Pritchard et al., 2000). Barley is known to have a clear population structure with distinct sub-populations due to differences in row-type, i.e., two-row and six-row, as well as in seasonal growth habit, i.e., spring and winter (Malysheva-Otto et al., 2006; Rostoks et al., 2006; Hamblin et al., 2010; Rajala et al., 2016). Several statistical models to account for the strong population structure, hence reducing the inflation of false positive associations, have been developed (Price et al., 2006; Kang et al., 2008; Stich and Melchinger, 2009).

Recently, 15 QTLs for powdery mildew resistance were identified by GWAS in a wild barley collection, out of which seven were novel QTLs for powdery mildew resistance (Ames et al., 2015). Wild barley, *H. v.* ssp. *spontaneum*, is the progenitor of cultivated barley and can be used as a source of resistance. However, to introduce such novel QTL from wild barley to cultivated barley takes many back-crossing generations. Nevertheless, this demonstrates the potential of GWAS in detection of new markers for resistance traits.

In the present study, the aim was to identify SNP markers significantly associated with powdery mildew resistance loci in a Nordic spring barley panel using GWAS. The Nordic spring barley panel consisted of 169 breeding lines and cultivars provided by five Nordic plant breeding entities. Markers linked with known and novel powdery mildew resistance genes enable breeders to test for and combine the resistance genes in their breeding lines.

## Materials and Methods

### Nordic Spring Barley Panel

The association mapping panel consisted of 169 spring *H. vulgare* L. ssp. *vulgare* advanced breeding lines and cultivars originating from Boreal Plant Breeding (27), Graminor Breeding AS (28), Agricultural University of Iceland, LBHI (26), Lantmännen Lantbruk, LSW (28), Nordic Seed (28), and Sejet Planteforaedling I/S (30). Out of the 169 lines, 124 lines were two-rowed and 45 six-rowed. Further description of the lines can be found in Bengtsson et al. (2017).

### Field Trials and Phenotypic Evaluation

The spring barley panel was evaluated for powdery mildew resistance in naturally infected fields for three consecutive years and with one or two observations over time per year at the following locations: southern Sweden at Svalöv (2012, 2; 2013, 2; 2014, 2) and Bjertorp (2013, 1), Denmark at Dyngby (2012, 1; 2014, 2) and Horsens (2013, 1), and Norway at Værnes (2014, 1). The field trials conducted in Sweden were sown in two replicates using a randomized complete block design. In Dyngby three replicates with three line rows, 1 m per replicate were used. At Horsens the trial was sown in four replicates with a plot size of 1.5 m × 2.5 m in an alpha lattice design with incomplete blocks. A hill plot system in an alpha lattice design with 50 seeds per plot in two replications, were used in Norway. The field designs were selected based on each breeding entities’ daily practices and field availability.

Statistical analyses of the phenotypic evaluations using the ordinal 1–9 disease rating scale (1 = minimum susceptibility, 9 = maximum susceptibility) were made in Minitab, release 16 (Minitab Inc., 2010). Mean values from replicates of a certain line, observation time, field location, and year were used for calculations of the frequency distribution whereas mean values from replicates of a certain line were used for calculations of the Spearman’s correlation coefficients.

### Genotyping

DNA was extracted from 2 weeks old seedlings, using a CTAB (Cetyl Trimethyl Ammonium Bromide) method as described earlier by Orabi et al. (2014). The spring barley panel was genotyped using 48 SSR markers evenly distributed over all chromosomes and the barley iSelect SNP chip based on the Illumina Infinium 9K assay as described in Bengtsson et al. (2017). The SNP genotyping of the lines were outsourced to Trait Genetics (Gatersleben, Germany).

#### *mlo* Genotyping

The presence of *mlo* alleles in the spring barley panel was evaluated according to Piffanelli et al. (2004) for *mlo-*11 (MITE-Fw 5′-CTCCATTTGACTTGACTCG-3′, MITE-Rw 5′-CATGCATGGTTATTGTAAGC-3′) and by designing allele specific primers for *mlo-*5 and *mlo-*9 (*mlo*-5 and *mlo*-9 Fw 5′-GTAGCGTGCGCTTTCTTTTT-3′, *mlo*-5 Rw 5′-GCACCCCTTTTTTGTCCGA[C/T]-3′ and *mlo*-9 Rw 5′-GGCGTCTCCGGCAGCTCCC[G/A]-3′) according to Büschges et al. (1997). PCR amplifications were performed in Thermo-Fast 96-well plates in a final volume of 10 μl containing the following components: 100 ng/μl template DNA, 1x key buffer, 1.5 mM MgCl_2_, 200 μM dNTPs, 250 nM each forward and reverse primer and 0.25 U Taq-polymerase. Amplifications were carried out using a GeneAmp PCR System 2700 thermal cycler (Applied Biosystems, Foster City, CA, United States) with the following program: an initial denaturation step at 94°C for 60 s, followed by 18 touchdown cycles (decrementing 0.5°C per cycle) consisting of a denaturation step of 60 s at 94°C, an annealing step between 64 and 55°C for 30 s and an elongation step at 72°C for 60 s. Next, 20 cycles with a denaturing step of 60 s at 94°C, annealing step of 60 s at 55°C and an elongation step of 60 s at 72°C were performed. The final elongation step was carried out at 72°C for 5 min. The PCR products were evaluated on a 1.5% agarose gel with expected product sizes of 530 bp for *mlo*-11, 238 bp for *mlo*-5 and 263 bp for *mlo*-9.

### Population Structure and Linkage Disequilibrium (LD)

Analyses of population structure based on a Bayesian clustering approach (STRUCTURE v.2.3.4, Pritchard et al., 2000), linkage disequilibrium (allele frequency correlation, r^2^) estimates between the SNP marker pairs using the full matrix option (Tassel 3.0 software^1^) and LD decay within this spring barley panel were performed and reported by Bengtsson et al. (2017).

Due to the non-normal data distribution we have, and to compare powdery mildew severity between structure groups, Kruskal–Wallis analysis was performed in Minitab, release 16 (Minitab Inc., 2010). Kruskal–Wallis analysis utilizes the median values and uses the *p*-value to determine whether any of the differences between the medians are statistically significant. The calculated mean from the powdery mildew observations and replicates of a certain line, using the ordinal 1–9 scale, were input data for calculations of the median value.

### Genome-Wide Association Study (GWAS) of the Nordic Spring Barley Panel

Genome-wide association study based on the SNP markers was performed using Tassel version 5 (Bradbury et al., 2007). The SNPs were filtered for MAF ≤ 0.05 resulting in 5556 SNPs left for GWAS analyses. Nine models comprising both general linear models (GLMs) and mixed linear models (MLMs) were tested to account for the strong population structure, hence reducing the inflation of false positive associations. The different models included covariates from principal component analysis (PCA) obtained from Tassel, ancestry coefficient data (Q matrix) obtained from STRUCTURE, genetic distance (D) matrix and the relative kinship matrix (K) obtained from Tassel. D matrices from the SSR and the SNP markers were calculated using an in-house program written in VBA (Visual Basic for Applications) and implemented in Microsoft Excel 2007 (Microsoft, Redmond, WA, United States). The program utilizes R language software v.2.14.2 (R Development Core Team, 2012), which includes the modern applied statistics with S-plus (MASS) package (Venables and Ripley, 2002). Further details about the in-house program can be found in the paper by Ingvordsen et al. (2015).

The following GLM methods were tested: (i) Naïve (GLM without any correction for population structure); (ii) SNP distance (GLM, correction with D_SNP_ matrix); (iii) SNP distance – PCA (GLM, correction with D_SNP_ matrix and PCA covariates); (iv) SNP distance – SSR (GLM, correction with D_SNP_ matrix and Q_SSR_ matrix); (v) SNP distance – SNP (GLM, correction with D_SNP_ matrix and Q_SNP_ matrix) and the tested MLM methods were: (i) EMMA [efficient mixed-model association; MLM, correction with kinship (K) matrix]; (ii) EMMA – PCA (MLM, correction with K-matrix and PCA covariates); (iii) EMMA – SSR (MLM correction with K-matrix and Q_SSR_-matrix); (iv) EMMA – SNP (MLM correction with K-matrix and Q_SNP_-matrix).

A combined quantile–quantile plot for all models and methods, showing the relative distribution of the observed -log10 (*p)*-values for each marker – trait association compared with the cumulative were plotted in R. When selecting the best model and method the following criteria were considered (i) least deviation from the expected *p-*values, (ii) highest number of groups, (iii) high heritability, (iv) the lowest compression value, and (v) the lowest variance error. Association analysis between SNP markers and mildew infection was made using the model best fitting the criteria. The critical *p*-values for assessing the significance of associations in the naive model were corrected for multiple comparisons based on the Bonferroni method where the adjusted *p*-value = -log10 (α/n), α = significance level and n = number of observations (Bonferroni, 1935, 1936). Thus the Bonferroni adjusted cut-off for accepting associations were set to -log10 (*p)-*value ≥ 5.0 which corresponds to an experiment wise error rate of 0.05.

Kruskal–Wallis analyses were performed for the powdery mildew comparison between markers identified in the GWAS analysis. The input data for these calculations were the calculated mean score from replicates of a certain line, observation time, location, and year (*N* = 169).

## Results

### Powdery Mildew Evaluation

**FIGURE 1:**
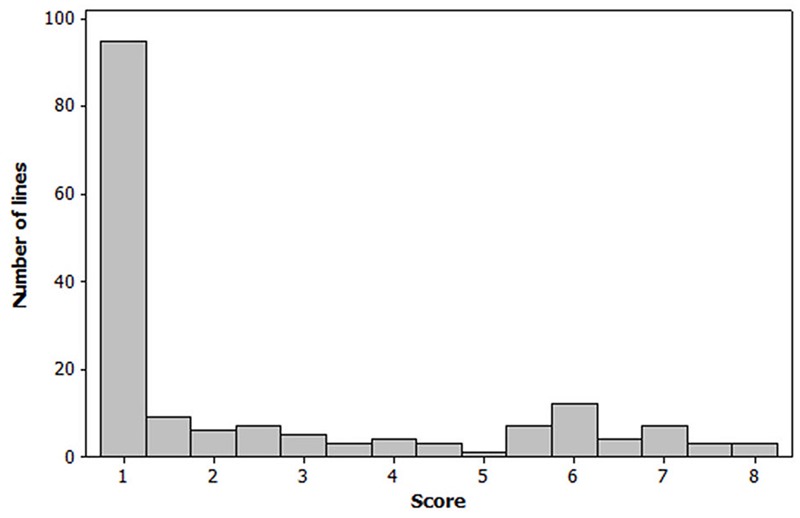
Frequency distribution of the barley lines based on their powdery mildew mean scores in the Nordic field trials 2012, 2013, and 2014. Each mean value is based on replicates of a certain line, observation time, location, and year (*N* = 169). Infection was scored on a 1–9 scale, where 1 = not infected and 9 = heavily infected.

The Spearman correlation analysis revealed a high correlation between all powdery mildew evaluations performed in the field during the 2012, 2013 and 2014 growing seasons, coefficients ranging between 0.77 and 0.96 (**Table 1**). The distribution of the phenotypic values for powdery mildew resistance was skewed toward resistant reactions and the residuals did not follow a normal distribution (**Figure 1**). The mean values and range scores of the powdery mildew infection within each location for every observation and year can be found in Supplementary Table 1.

**Table 1:** Spearman’s correlation coefficients^∗^ of powdery mildew mean scores in the Nordic field trials 2012–2014.

	Dyngby 2012 (1)	Svalöv 2012 (1)	Svalöv 2012 (2)	Svalöv 2013 (1)	Svalöv 2013 (2)	Bjertorp 2013 (1)	Horsens 2013 (1)	Svalöv 2014 (1)	Svalöv 2014 (2)	Værnes 2014 (1)	Dyngby 2014 (1)	Dyngby 2014 (2)
Dyngby 2012 (1)	1											
Svalöv 2012 (1)	0.86	1										
Svalöv 2012 (2)	0.87	0.93	1									
Svalöv 2013 (1)	0.88	0.85	0.82	1								
Svalöv 2013 (2)	0.83	0.84	0.81	0.89	1							
Bjertorp 2013 (1)	0.87	0.87	0.87	0.85	0.87	1						
Horsens 2013 (1)	0.92	0.91	0.90	0.87	0.88	0.90	1					
Svalöv 2014 (1)	0.80	0.79	0.81	0.80	0.80	0.81	0.83	1				
Svalöv 2014 (2)	0.87	0.88	0.86	0.91	0.91	0.89	0.91	0.84	1			
Værnes 2014 (1)	0.87	0.87	0.85	0.88	0.88	0.87	0.90	0.81	0.91	1		
Dyngby 2014 (1)	0.87	0.85	0.82	0.90	0.84	0.82	0.86	0.80	0.87	0.90	1	
Dyngby 2014 (2)	0.83	0.81	0.80	0.85	0.81	0.80	0.86	0.79	0.85	0.88	0.94	1

*^∗^The correlation coefficients are based on mean values from the replicates of a certain line (df = 167). Figures given in parenthesis are representing the first and second observation. All correlations were statistically significant at *p*-value ≤ 0.05 (Minitab v. 16).*

### Genotyping

Out of the 7,842 high-confidence SNPs, derived from expressed genes, on the barley iSelect SNP chip based on the Illumina Infinium 9K assay, 6,208 SNPs were polymorphic. The polymorphic information content (PIC) values ranged between 0.26 (1H) and 0.31 (6H) with an average of 0.28 (**Table 2**). The marker coverage for the seven chromosomes varied between 0.17 and 0.28 (**Table 2**). The genotyping based on the SSR markers resulted in 234 scorable loci and an average PIC value of 0.46. More detailed information regarding the genotyping, as well as results from the comparison of the two marker systems, can be found in Bengtsson et al. (2017).

**Table 2:** SNP coverage, polymorphism (PIC) and distribution across chromosomes^a^.

Chromosome	Length (cM)	No. of markers	Marker coverage	Average PIC
1H	140.5	506	0.28	0.26
2H	160.3	814	0.20	0.30
3H	173.2	785	0.22	0.29
4H	123.3	615	0.20	0.29
5H	196.1	1058	0.19	0.30
6H	129.4	752	0.17	0.31
7H	166.6	698	0.24	0.30
Total	1089.4	5228	0.21	0.28

*^a^Only markers with a known chromosomal position included.*

#### *mlo* Evaluation

Lines tested positive with primers for any of the three *mlo* alleles *mlo*-5, *mlo*-9, and *mlo*-11 were considered as *mlo* lines. Out of the 169 lines, 109 were found to be *mlo* lines. There was a large difference between the two row types, where an *mlo* allele was found in 78% of the two-rowed lines but in just 27% of the six-rowed lines.

### Powdery Mildew within the Structure Groups

**FIGURE 2:**
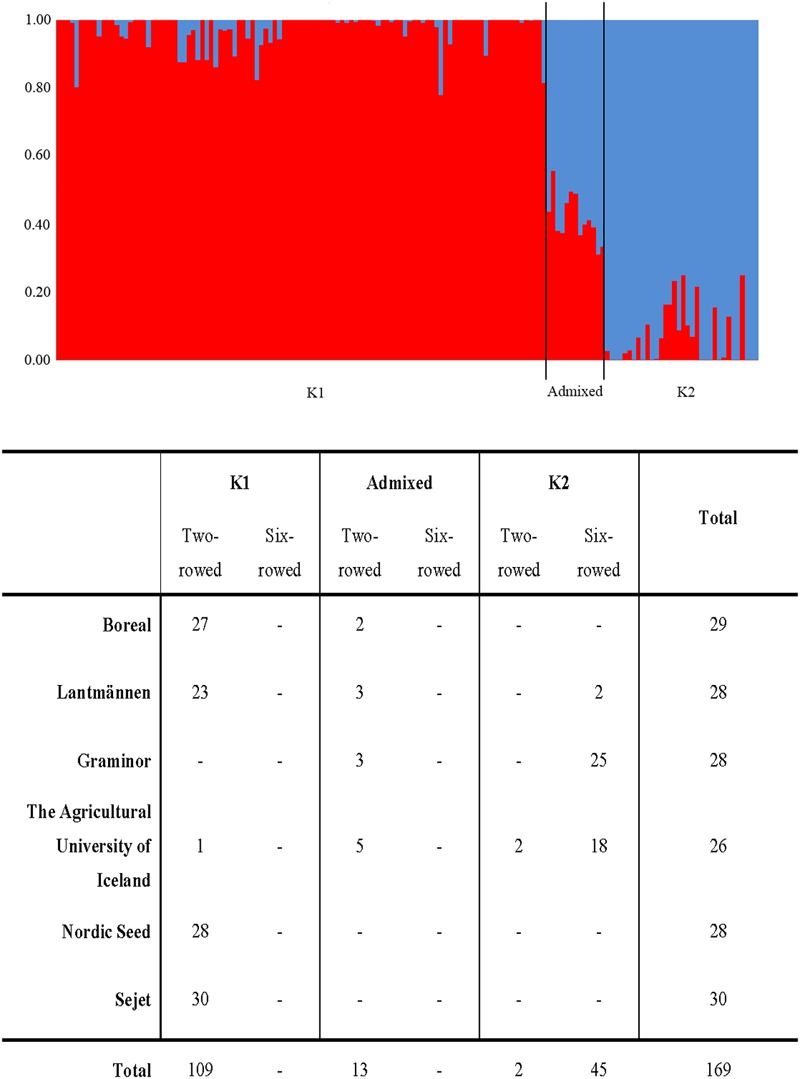
Sub-groups in the Nordic spring barley panel according to STRUCTURE software analysis. The table shows number of lines in each sub-group from each breeding entity, split on two-rowed and six-rowed genotypes.

The Bayesian clustering analysis showed population sub-structuring within the spring barley panel; group K1 (*N* = 109) consisting only of two-rowed lines, K2 (*N* = 47) consisting mainly of six-rowed lines and admixed (*N* = 13) (**Figure 2**). The admixed group is a small group of two-rowed lines, from the northern parts of the Nordic region. Breeder origin and row type of each line have been reported earlier by Bengtsson et al. (2017). The highest powdery mildew infection mean was found in sub-groups admixed and K2 whereas the lowest infection rate was found in sub-group K1 (**Table 3** and **Figure 2**). Kruskal–Wallis analysis revealed that the K1 median was significantly different (*p-*value ≤ 0.05) from those of both K2 and the admixed group with a negative *Z*-value which indicates that the K1 group’s average rank is less than the overall average (**Table 3**). No significant difference was observed between K2 and the admixed group. The proportion of lines with *mlo* alleles in K1 and K2 were 88 and 26%, respectively, whereas no *mlo* alleles were found in the admixed group.

**Table 3:** Powdery mildew (Pm) infection rates of the sub-groups in the Nordic spring barley panel, consisting of 169 lines, based on data from the five different field locations during 2012–2014.

	No. of	Median	Average	
Sub-groups	observations	Pm score^∗^	rank	*Z*
K1	872	1.0^a^	501.6	-22.20
K2	376	5.0^b^	963.9	16.80
Admixed	104	5.0^b^	1103.9	11.62
Overall	1353		676.5	

*^∗^Input data were the calculated mean from replicates of a certain line (and observation time if there was more than one scoring per season) for each location and year (*N* = 1353). Different lower case letters indicate statistically significant differences (*p-*value ≤ 0.05) using pair-wise Kruskal–Wallis comparisons. Average Rank = the average of the ranks for all observations within each sample. *Z* = value of how the average rank for each group compares to the average rank of all observations.*

### Genome-Wide Association Analysis of the Nordic Spring Barley Panel

#### Selection of Model

**FIGURE 3:**
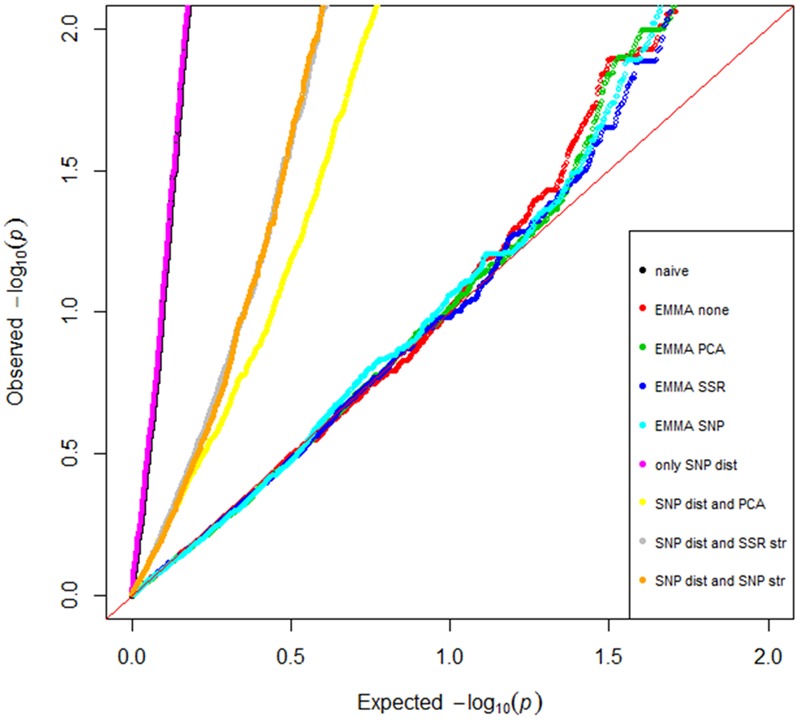
Model comparisons for the genome-wide association study. Quantile–quantile plot of the expected versus the observed distribution of *p*-values were computed from 5,556 SNP markers for the different association models.

The QQ-plot (**Figure 3**), showing the relative distribution of observed -log10 (*p)-*values for every model in comparison with the cumulative distribution, revealed numerous spurious observations when applying the naïve model and the four GLM using the D_SNP_ matrix. Among the GLM models the model including the D-matrix and PCA covariates from the SNP data was best in correcting for the structure. However, the four MLM using the K matrix were found to better account for the observed population structure and showed a similar distribution. Thus these EMMA models were further compared based on number of groups, compression, heritability, explained genetic variance, variance error and minus two log likelihood function value (-2LnLk). The number of groups and compression level were 169 and 1, respectively, for all EMMA models. The EMMA-none model had highest heritability (0.90), the highest genetic variance (1.09), and lowest error (0.13) and was therefore used in all GWAS analysis.

#### Associations with Powdery Mildew Resistance

**FIGURE 4:**
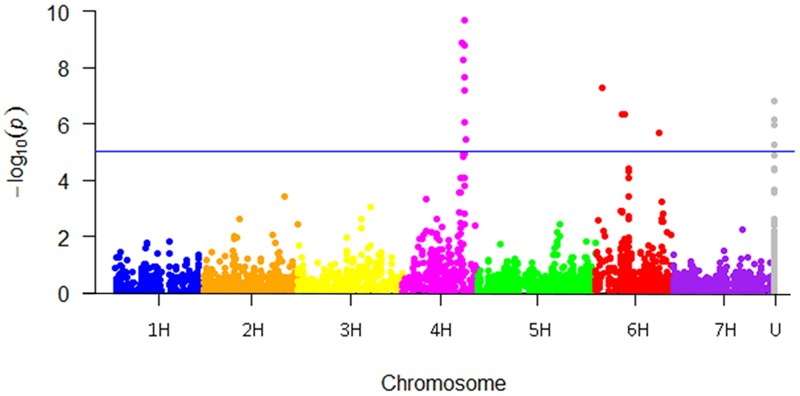
Manhattan plot showing the genome-wide association study (GWAS) results for powdery mildew in the Nordic spring barley panel. The Bonferroni adjusted cut-off for accepting associations were set to –log10 (*p)-*value ≥ 5.0 corresponding to an experiment wise error rate of 0.05. U = genetic chromosomal location currently unknown.

Genome-wide association between the SNP markers and powdery mildew resistance was performed based on overall mean values from all locations. A Manhattan plot showing the -log10 (*p*)-values for the markers according to chromosomal position, except four cases where the genetic chromosomal positions are unknown (“U” in **Table 4**), are shown in **Figure 4**. A total of 18 significant SNP marker-trait associations (*p-*value ≤ 0.05, Bonferroni corrected) were found, that could be grouped into one QTL on 4H (99–104 cM) and three QTLs on 6H (11, 45–49, and 106 cM). The effect of the most significant marker within each QTL varied between 2.0 and 2.9 (on a disease scale from 1 to 9) (**Table 4**).

**Table 4:** Significant markers associated with powdery mildew resistance using the EMMA model in the Nordic spring barley panel.

SNP	Chromosome	Position	*p*-value	-log10(p)	Marker *R*^2^	Effect	QTL
SNP4H_1	4H	102	2.03E-10	9.69	0.27	2.8	QPM.PPP-4H
SNP4H_2	4H	99	1.28E-09	8.89	0.25	2.0	
SNP4H_3	4H	101	1.65E-09	8.78	0.24	2.1	
SNP4H_4	4H	102	1.65E-09	8.78	0.24	2.1	
SNP4H_5	4H	101	5.21E-09	8.28	0.23	1.9	
SNP4H_6	4H	102	2.13E-08	7.67	0.21	1.7	
SNP4H_7	4H	101	6.56E-08	7.18	0.19	2.0	
SNP4H_8	U^a^	U^a^	1.55E-07	6.81	0.18	1.5	
SNP4H_9	4H	103	8.36E-07	6.08	0.16	1.9	
SNP4H_10	U^a^	U^a^	1.10E-06	5.96	0.15	1.1	
SNP4H_11	4H	104	3.57E-06	5.45	0.14	1.3	
SNP4H_12	4H	104	3.57E-06	5.45	0.14	1.3	
SNP4H_13	U^a^	U^a^	5.40E-06	5.27	0.13	-1.3	
SNP6H_1_1	6H	11	5.45E-08	7.26	0.19	-2.8	QPM.PPP-6H-1
SNP6H _2_1	6H	45	4.39E-07	6.36	0.16	2.9	QPM.PPP-6H-2
SNP6H _2_2	6H	49	4.39E-07	6.36	0.16	2.9	
SNP6H _3_1	6H	106	2.08E-06	5.68	0.14	2.0	QPM.PPP-6H-3
SNP6H _4_1	U^b^	U^b^	7.31E-07	6.14	0.16	2.4	

*U = genetic chromosomal location currently unknown. ^a,b^Chromosome location of SNP markers assigned to 4H and 6H, respectively, based on blast information at plant ensembl: http://plants.ensembl.org/Hordeum_vulgare/Info/Index. *R*^2^= sum of the R square, which quantifies goodness of fit in a range between 0.0 and 1.0.*

Blast information^2^ revealed that the most significant marker, SNP4H_1 marker in QTL QPM.PPP-4H on chromosome 4H, and the *mlo* gene are physically close (∼3 Kbp, data not shown). A significantly lower (*p-*value ≤ 0.05) median mildew score was found among lines with the positive allele of the SNP4H_1 marker for QPM.PPP-4H and the *mlo* lines compared to the lines having the negative allele of SNP4H_1 and the non-*mlo* lines, respectively (**Table 5**). There was no difference in the median mildew score between lines with the resistance allele of marker SNP4H_1 and the lines carrying one of the *mlo* resistance alleles (**Table 5**). All *mlo* lines were found to have the positive allele of the SNP4H_1 marker and these lines had an average powdery mildew score of 1.2. The 15 non-*mlo* lines with the positive allele for SNP4H_1 had a median mildew score of 3.1.

**Table 5:** Powdery mildew (Pm) comparisons between the presence of *mlo* alleles and the most significant QPM.PPP-4H marker, physically close to *mlo.*

Marker (allele)	No. of lines	Median Pm score^∗∗^	Average rank	*Z*
SNP4H_1 (A)	45	6.9	145.9	9.75
SNP4H_1 (G)	124	1.0	62.9	-9.75
*Non-mlo*^∗^	60	6.3	138.1	10.47
*mlo*^∗^	109	1.0	55.8	-10.47

*^∗^Determined based on presence of any of the *mlo-*5, *mlo-*9, or *mlo*-11 alleles. The comparisons were statistically significant at *p*-value ≤ 0.05 (Pairwise Kruskal–Wallis comparison, Minitab v. 16). ^∗∗^The median values are based on the calculated mean from replicates of a certain line, observation time, location, and year (*N* = 169). Average Rank = the average of the ranks for all observations within each sample. *Z* = value of how the average rank for each group compares to the average rank of all observations.*

Almost all *mlo* lines were found to also have the positive alleles of the most significant markers within QPM.PPP-6H-1, QPM.PPP-6H-2, and QPM.PPP-6H-3 (data not shown). The top NCBI BLASTn hit^3^ for SNP6H_2_2 was predicted to be in the gene for 26S proteasome regulatory subunit, RPN1. For SNP6H_2_1 the top hit was an mRNA sequence from a *H. v.* ssp. *vulgare* cDNA clone: FLbaf61b05, with unknown function. No BLASTn hits for the other markers on 6H were found. Since the GWAS results revealed that the two markers in QPM.PPP-6H-2 had equal effect and significance level, the SNP6H_2_2 marker was chosen to be used for further marker comparisons.

The positive alleles of marker: SNP6H_1_1, SNP6H_2_2, and SNP6H_3_1 on 6H were associated with a significantly reduced (*p-*value ≤ 0.05) median powdery mildew score in the non-*mlo* lines; from 7.7 to 4.9, 7.0 to 3.5 and 7.6 to 4.9, respectively (**Table 6**). Lines having all the positive alleles for the SNP6H_1_1, SNP6H_2_2, and SNP6H_3_1 marker had the lowest median powdery mildew score (haplotype CGT; 1.0), which was significantly lower than the median powdery mildew score found among the lines having all the three negative alleles (haplotype TAC; 7.7) (**Table 7**). The lowest powdery mildew median scores were found for the haplotypes including the positive allele of SNP6H_2_2 (CGC, 1.4; CGT, 1.0; TGT, 3.7).

**Table 6:** Allele distributions of the most effective markers in QPM.PPP-6H-1 to 3 in non-*mlo* lines.

		Non-*mlo* lines	
QTL	Marker (allele)	No. of lines	Median Pm score^∗^
QPM.PPP-6H-1	SNP6H_1_1 (C)	31	4.9^b^
	(T)	29	7.7^a^
QPM.PPP-6H-2	SNP6H_2_2 (G)	18	3.5^b^
	(A)	42	7.0^a^
QPM.PPP-6H-3	SNP6H_3_1 (T)	33	4.9^b^
	(C)	27	7.6^a^

*^∗^The median values are based on the calculated mean from replicates of a certain line, observation time, location, and year (*N* = 60). Different lower case letters indicate statistically significant differences (*p-*value ≤ 0.05) using pair-wise Kruskal–Wallis comparisons.*

**Table 7:** Haplotype effect of the most effective markers in QPM.PPP-6H-1 to 3 on the powdery mildew (Pm) infection.

No. of lines	SNP6H_1_1	SNP6H_2_2	SNP6H_3_1	Median Pm score^∗^
1	**C**	A	C	7.6^abc^
14	**C**	A	**T**	5.7^b^
3	**C**	**G**	C	1.4^b^
121	**C**	**G**	**T**	1.0^a^
25	T	A	C	7.7^c^
3	T	A	**T**	8.1^bc^
2	T	**G**	**T**	3.7^abc^

*^∗^The median values are based on the calculated mean from replicates of a certain line, observation time, location, and year (*N* = 169). Positive alleles are marked in bold. Different lower case letters indicate statistically significant differences (*p-*value ≤ 0.05) using pair-wise Kruskal–Wallis comparisons.*

The positive allele of the most significant marker in QPM.PPP-4H and QPM.PPP-6H-2, reduced the mean infection scores by 25 and 35% among the lines only having the positive allele for QPM.PPP-4H (three lines) or QPM.PPP-6H-2 (five lines), respectively, whereas a reduction of the mean powdery mildew infection score by 79% was found among the lines having both of the positive alleles (121 lines).

## Discussion

This panel of advanced breeding lines and cultivars consists of two genetically distinct groups each with a common ancestry, K1 and K2, and one admixed group (Bengtsson et al., 2017). The present study revealed that the proportion of lines with *mlo* alleles was 88% in K1 (only two-rowed lines) and 26% in K2 (mostly six-rowed lines), whereas no *mlo* alleles were found in the admixed group consisting of cultivars and breeding lines from the northern part of the Nordic region. This difference explains the low powdery mildew infection mean observed in K1 compared to the other two groups. A high proportion of lines with *mlo* alleles among the two-rowed lines is expected since the risk of powdery mildew infection is higher in the southern region with its milder climate, and where mostly two-rowed barley is cultivated (Hovmøller et al., 2000).

Due to the strong population structure observed in this spring barley panel, several models were tested to control for structure. To find the best model, the naïve GLM model was compared with variants including the SNP D-matrix and with EMMA-none with variants for corrections (**Figure 3**). The EMMA-none model was superior to all methods tested in controlling the strong population structure in this spring barley panel (**Figure 3**), as well as for controlling structure in a world-wide spring barley collection (Pasam et al., 2012). The EMMA-none model is faster to compute compared to the linear models and has the advantage that it does not require any additional matrix for calculation.

Using the EMMA-none model and field data, consistent between observation times, years and locations (**Table 1**), 18 significant marker-trait associations were found for powdery mildew with an estimated marker effect of the most significant marker within each QTL varying between 2.0 and 2.9 (on a disease scale from 1 to 9) (**Table 4**). Out of these, four significant marker-trait associations were found for previously unmapped markers, however three of them could be assigned to chromosome 4H and one to 6H using blast information at plant ensembl^4^.

The three markers on 4H were assigned to QPM.PPP-4H. In total 13 significant SNP markers were found within a QTL (QPM.PPP-4H) on 4H, with an average mildew score reduction of 2.8 for the most significant marker-trait association and locating physically close (∼3 Kbp) to the previously identified *mlo* locus (Piffanelli et al., 2004). This was expected, since 64% of the barley lines included in this study contains one of the *mlo* resistance alleles *mlo-*5, *mlo-*9 (Büschges et al., 1997) or *mlo-*11 (MITE) (Piffanelli et al., 2004). Interestingly, there was no difference in the median mildew score between the lines having the positive allele for the QPM.PPP-4H, SNP4H_1 marker and the *mlo* lines (**Table 5**), which shows that the markers within QPM.PPP-4H can be good candidates for future use in marker-assisted selection for powdery mildew resistance in barley. The SNPs found within QPM.PPP-4H can easily be converted to easy-to-use DNA markers such as competitive allele-specific PCR (KASP) markers (Semagn et al., 2014), which enables fast and high-throughput analysis compared to the more time-consuming agarose-based analysis associated with the MITE markers for *mlo* (Piffanelli et al., 2004). However, there were 15 non-*mlo* lines with the positive allele for SNP4H_1 that had a low median mildew score, but not as low as for the *mlo* lines. Still the benefits with a high-throughput and faster analysis for screening of powdery mildew resistance based on the SNP4H_1 marker are higher than the disadvantage of at the same time selecting a few lines without *mlo* with a somewhat higher but still low average mildew score.

Four significant marker-trait associations were also located on chromosome 6H and assigned to two QTLs on the short arm (QPM.PPP-6H-1, QPM.PPP-6H-2) and one on the long arm (QPM.PPP-6H-3). The significant marker-trait association identified on 6H at 11 cM, QPM.PPP-6H-1, was within the same region (19 cM) as a QTL (Bgh-qtl-6H-bPb-8473) recently identified to be associated with powdery mildew resistance in wild barley (Ames et al., 2015). Two significant marker-trait associations were found located on 6H at 45 and 49 cM within QPM.PPP-6H-2, which had a similarly strong effect as the QTL QPM.PPP-4H. This could be due to the strong LD found between the markers within QPM.PPP-4H and markers within QPM.PPP-6H-2 (r^2^ between 0.5 and 0.8, data not shown) which most likely is caused by the strong population structure. However, when comparing the haplotype effect of the most significant marker in QPM.PPP-4H and QPM.PPP-6H-2, the mean infection scores were reduced considerably more among the lines having both of the positive alleles compared to the lines only having the positive allele for either of the two. Altogether, this indicates that QPM.PPP-6H-2 adds to the QPM.PPP-4H effect.

When comparing the haplotypes for the most effective markers in QPM.PPP-6H-1 to 3, the lowest powdery mildew median scores were also found in the haplotypes including the most effective marker within QPM.PPP-6H-2 (**Table 7**). The spring barley panel appears to suffer from the skewness due to the large number of lines carrying *mlo* resistance allele and this may affect the GWAS results. However, the mildew scores for the different haplotypes indicate that we may be dealing with a new QTL. No other QTL for powdery mildew resistance has so far, to our knowledge, been reported within the location of QPM.PPP-6H-2; hence this could be a novel QTL for powdery mildew resistance. The top NCBI BLASTn hit for SNP6H_2_2 within QPM.PPP-6H-2 is predicted to be the protein 26S proteasome regulatory subunit, RPN1. RPN1a has previously been reported to be required for innate immunity and mildew-induced cell death in *Arabidopsis* (Yao et al., 2012) as well as to negatively control ABA signaling and to function during embryogenesis and stress responses in *Arabidopsis* (Brukhin et al., 2005; Yu et al., 2016). Another significant marker-trait association QPM.PPP-6H-3 was found on the long arm of 6H at 106 cM which is close to the powdery mildew resistance QTL, Rbgq (Aghnoum et al., 2010). Possibly it marks the same QTL.

Even though *mlo*-resistance is common in barley cultivars and valued among growers, there are reports showing that it may lead to an increased susceptibility to necrotrophic and hemi-biotrophic pathogens (Kumar et al., 2001; Brown and Rant, 2013; McGrann et al., 2014). A negative correlation between powdery mildew and Ramularia infestation found also in our material may be in support of this (data not shown). In addition, *mlo*-resistance has been shown to be accompanied with deleterious pleiotropic effects such as development of spontaneous leaf cell death lesions in the absence of pathogens, leading to some reduction in yield (Kjaer et al., 1990; Thomas et al., 1998). This makes the localization of the markers for powdery mildew resistance on chromosome 6H very valuable for breeders when breeding for multiple disease resistance in barley in the Nordic region, even though the mildew reducing effect is not as dramatic as with *mlo*.

In a previous study of a world-wide collection of spring barley, the spike morphology trait ‘row-type’ (two-rowed spike vs. six-rowed spike) were used as a proof-of-concept for the GWAS and a total of 34 marker-trait associations including associations with known spike morphology loci like *vrs1, vrs2, vrs3, vrs4*, and *int-c* were identified (Pasam et al., 2012). The same analysis was performed in our study to evaluate the EMMA model used in the GWAS for powdery mildew resistance. In contrast to the results reported by Pasam et al. (2012), only two significant marker-trait associations for spike morphology, one on 2H (74 cM) and one on 4H (26 cM), were identified here. The two associations were concurrent with the previously identified major spike morphology loci *vrs1* and *int-c* (Komatsuda et al., 2007; Ramsay et al., 2011). One possible explanation for this difference between these two studies might be that the number of six-rowed lines used in the present study was less than half of the number of six-rowed lines used by Pasam et al. (2012). Another explanation could be the stricter threshold used in this study to claim significant associations. Nevertheless, the logarithmic values for the two associations detected here is substantially higher [-log10 (*p*) > 25] compared to the values for the spike morphology associations reported by Pasam et al. (2012). The two associations detected for spike morphology in our study demonstrate the stringency and accuracy of the GWAS analysis. However, it is important to consider that while reducing the number of false-positives, the number of false negatives may increase. It has earlier been shown that increasing the stringency of the GWAS model in barley results in that a larger proportion of the trait variation is explained by the model itself rather than by genetic effects (Pasam et al., 2012). In our study the percentage of genetic trait variation detected (*R*^2^ values) for the corresponding SNPs were low, ranging from 0.13 to 0.27. Low *R*^2^ values have often been reported in studies of GWAS and the unexplained variation has been referred to as ‘unexplained missing heritability’ (Manolio et al., 2009). Several possible explanations for this ‘unexplained missing heritability’ has been proposed including; insufficient marker coverage, rare alleles (MAF < 5%) with a major effect excluded from the analysis, the trait depends on several genes/QTLs with small individual effects, inadequate statistical approaches to detect epistatic interactions and biased estimates of *R^2^* for individual SNPs caused by population stratification (Maher, 2008; Frazer et al., 2009; Manolio et al., 2009; Gibson, 2010; Hall et al., 2010). The strong population structure in the barley panel could be a possible explanation for the unexplained missing heritability observed in this study.

## Conclusion

Careful optimization of the model is needed to find an appropriate balance in terms of sensitivity and selectivity in GWAS of highly structured populations and inbreeding crops like barley. In this study the use of the best model to account for population structure resulted in the localization of three presumably known QTLs for powdery mildew resistance and the detection of one, to our knowledge, novel QTL on chromosome 6H. After validation the corresponding SNPs could be exploited for marker-assisted selection and for stacking of powdery mildew resistance genes in barley.

## Author Contributions

All authors were involved in the planning and design of the study, edited and approved the final manuscript. LR, TC, JDJ, and LK were responsible for the field experiments and phenotyping in the field. JO performed the genotyping of the SNPs and SSRs and the *mlo* evaluation. TB performed the GWAS and statistical analysis, wrote the manuscript, made the figures and finalized the tables. IÅ, OM, AJ, and JO assisted in the writing and interpretation of the data.

## Conflict of Interest Statement

The authors declare that the research was conducted in the absence of any commercial or financial relationships that could be construed as a potential conflict of interest.

## Footnotes

1 http://www.maizegenetics.net

2 http://plants.ensembl.org

3 https://blast.ncbi.nlm.nih.gov

4 http://plants.ensembl.org
