# Supplementary material for: A Novel QTL for Powdery Mildew Resistance in Nordic Spring Barley (Hordeum vulgare L. ssp. vulgare) Revealed by Genome-Wide Association Study
Source: Front Plant Sci. 2017 Nov 14;8:1954. doi: 10.3389/fpls.2017.01954 (PMC5694554; doi:10.3389/fpls.2017.01954)
Supplement: Supplementary file 1 [file Table_1.DOCX]

**Supplementary Table 1** Powdery mildew means and range scores in the Nordic field trials 2012 – 2014

|  | **Dyngby**  **2012**  **(1)** | **Svalöv 2012**  **(1)** | **Svalöv 2012**  **(2)** | **Svalöv 2013**  **(1)** | **Svalöv 2013**  **(2)** | **Bjertorp 2013**  **(1)** | **Horsens 2013**  **(1)** | **Svalöv 2014**  **(1)** | **Svalöv 2014**  **(2)** | **Værnes 2014**  **(1)** | **Dyngby**  **2014**  **(1)** | **Dyngby**  **2014**  **(2)** |
| --- | --- | --- | --- | --- | --- | --- | --- | --- | --- | --- | --- | --- |
|  |  |  |  |  |  |  |  |  |  |  |  |  |
| **Mean Pm infection** | 2.3 | 2.8 | 3.6 | 2.5 | 2.4 | 3.4 | 3.0 | 2.0 | 2.9 | 2.6 | 1.9 | 1.9 |
| **Range** | 5 | 7 | 8 | 8 | 8 | 8 | 8 | 6 | 8 | 8 | 6 | 6 |
| **Minimum** | 1 | 1 | 1 | 1 | 1 | 1 | 1 | 1 | 1 | 1 | 1 | 1 |
| **Maximum** | 6 | 8 | 9 | 9 | 9 | 9 | 9 | 7 | 9 | 9 | 7 | 7 |

Mean values were calculated from the replicates of a certain line (df =167). Figures given in parenthesis are representing the first and second observation. The calculations were performed using the Analysis ToolPak, a Microsoft Excel add-in program.
